# The importance of human interaction for curriculum and school life satisfaction among Japanese medical graduates: A web-based survey study

**DOI:** 10.1371/journal.pone.0319683

**Published:** 2025-04-24

**Authors:** Nobuyasu Komasawa, Masanao Yokohira

**Affiliations:** 1 Community Medicine Education Promotion Office, Faculty of Medicine, Kagawa University, Kagawa, Japan; 2 Department of Medical Education, Faculty of Medicine, Kagawa University, Kagawa, Japan; King Abdulaziz University Faculty of Medicine, SAUDI ARABIA

## Abstract

**Background:**

This study aimed to assess the curriculum and school life of Japanese medical students on the verge of graduation.

**Methods:**

A web-based questionnaire survey was conducted to gauge the goals of graduating medical students across various themes: Subjective academic achievement in each curriculum, Length of each curriculum, Timing for summative examination preparation, and Satisfaction with total medical school life, accomplishments, extracurricular activities, and friendships.

**Results:**

With a 67.8% response rate (80/118), the study found that subjective accomplishment in Clinical Clerkship (CC) was significantly lower than in other curriculums (P<0.05 each). Social medicine had significantly lower subjective accomplishment compared to basic medicine (P=0.040). Graduating students reported that the basic medicine curriculum was significantly longer than clinical medicine, CC, and general education (P < 0.05 each). Preparation timing for Pre-CC and Post-CC OSCE was significantly later compared to other summative tests (P < 0.05 each). Total satisfaction with medical school life correlated significantly with school friendships (P < 0.001), while subjective accomplishment and extracurricular activity did not.

**Conclusion:**

Graduating medical students express lower subjective accomplishment in CC compared to other curriculums, suggesting a need for CC content improvement. Additionally, the study highlights the significant role of school friendships in influencing total medical school life satisfaction.

## Introduction

For all educators and instructional physicians involved in educational practices to medical students, curriculum evaluation is an essential duty that should be undertaken continuously. Reflecting on one’s own educational practices and growing as an educator are crucial aspects, making curriculum evaluation important based on its outcomes [1,2]. If the evaluation aims to assess learner responses, conducting it immediately after educational activities is advisable. However, if assessing behavioral changes is the goal, long-term evaluation becomes more critical. In such cases, there is no definite answer to how much time should elapse before conducting the evaluation [3,4].

Combining short-term and long-term evaluations can lead to a more valid assessment, but this approach comes with increased costs and logistical challenges. Moreover, to incorporate evaluation results into medical education improvement, it is necessary to conduct evaluations and compile results before planning the next educational session [5].

From this perspective, the involvement of graduates in curriculum evaluation holds several significant implications. Graduates bring practical experience from working in healthcare settings. By evaluating the medical education curriculum, graduates provide insights from a practical standpoint, ensuring that the curriculum adequately covers the skills and knowledge required in real-life healthcare scenarios [6]. Graduates, actively engaged in the field, are better equipped to identify changes and advancements in healthcare. Their evaluation of the medical school curriculum helps in adapting it to meet the latest needs and trends in the medical field. Having experienced the curriculum as students, graduates can offer valuable perspectives on the educational process and course content [7]. This reflection from learner-centered view aids in creating a more student-friendly and effective learning environment.

Evaluating the alignment of the medical education curriculum with graduates’ occupational suitability ensures that future doctors acquire the necessary skills, enhancing their confidence and preparedness for medical education curriculum [8]. The participation of graduates is crucial for educational institutions to adapt to the realities of healthcare, ensuring that graduates acquire the skills demanded by the industry [9,10]. Feedback from their perspective is indispensable for improving the quality and adaptability of medical education. While curriculum evaluation by graduates is performed in various medical schools [11,12], most of them are after several years after graduation. Such time lag leads to the bias or fading of the memory in the responder. Furthermore, while extracurricular activity or school friend is also an essential factor related to curriculum, we also decided to assess the correlation among these factors.

Thus, we performed medical school curriculum evaluation survey from learner-centered view including surrounding factors to medical students who just took national exam for doctors are just to graduate during 1 month and evaluated the correlation.

In this study, we performed web-based survey on feedback about medical school curriculum and environment to medical students who are just to graduate.

## Materials and methods

### Ethical considerations

This research received approval from the Research Ethics Committee of the Faculty of Medicine, Kagawa University (No. 2023-204). As this Research Ethics Committee judged written informed consent is unnecessary, verbal informed consent was obtained from students by medical teacher and clerks also witnessed the process before the survey. All students were informed about the nature and purpose of the study and anonymity was guaranteed. Students were also informed that they had the opportunity to withdraw from the study if they notified the investigator within a week after they responded to the survey. We also emphasized that withdrawing from the study would not influence their academic outcomes in any way. Notably, all 6^th^ year medical students in Japan are over 23 years old; hence, the study did not involve any minors.

### Study population

**Fig 1:**
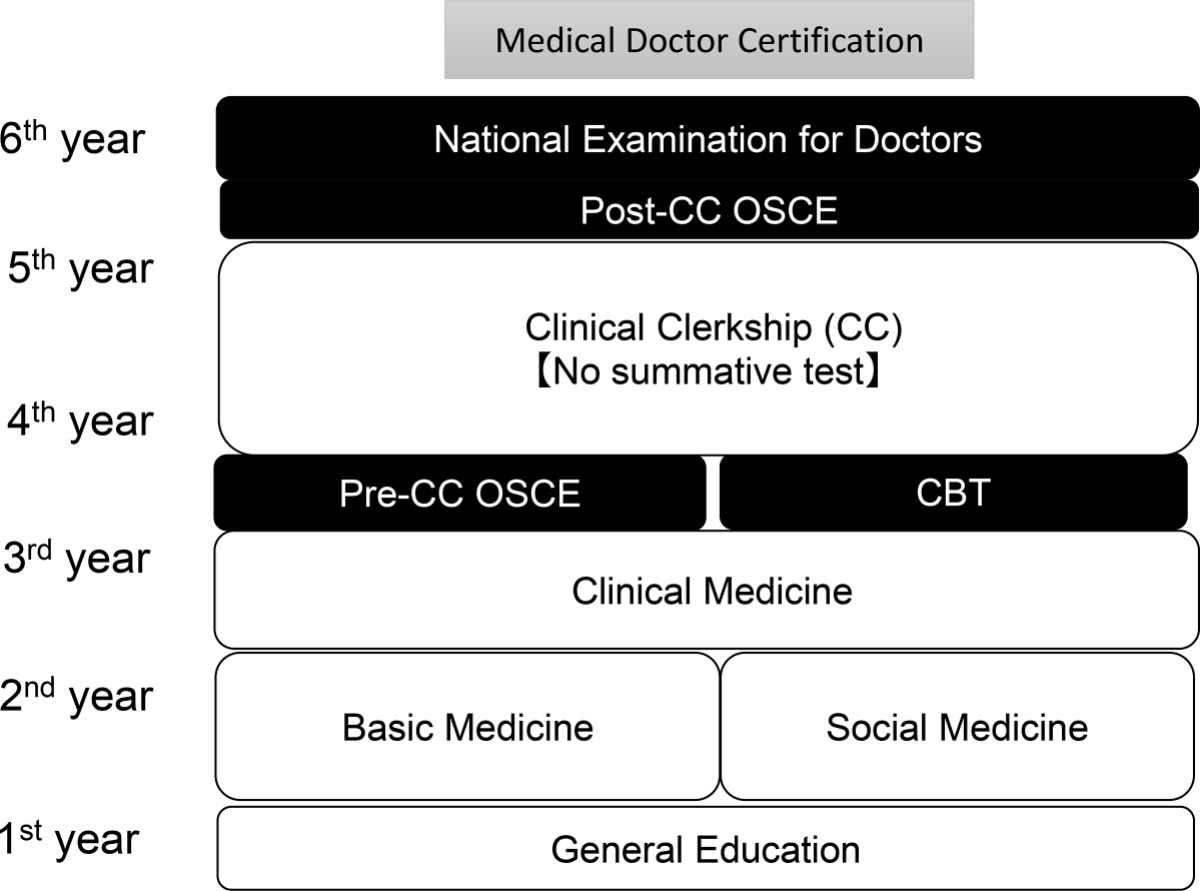
Curriculum course in our university and questionary timing on this survey.

We included for all medical students of 6^th^ year who are to graduate (118 people) as participants. Japanese medical schools usually consist of a 6-year study period. Students can enter medical school after graduating from high school and successfully passing an entrance exam. As with other medical schools in Japan, medical students at Faculty of Medicine, Kagawa University complete all general education, basic, social medicine in the first half of the medical education curriculum. In the 4^th^ grade, clinical medicine lectures were performed before beginning a CC [13,14]. After passing Computer based testing (CBT) and Objective Structured Clinical Education (OSCE), they start Clinical Clerkship in university or rural hospital for about 2 years [15,16]. In the 6^th^ grade, medical students undergo a Post-CC OSCE and take national examination for doctors (Fig 1).

### Study measures

After verbal informed consent to medical students during break time in the classroom, we sent the access site link via online communication system WebClass™ (Japan Data Pacific, Tokyo, Japan) which they can access easily [13].

We conducted a web-based questionnaire survey to evaluate goals of medical students regarding following themes.

Theme 1: Subjective academic achievement in each curriculum
Theme 2: Length of each curriculum
Theme 3: Timing to prepare for summative evaluation in each curriculum
Theme 4: General satisfaction for total curriculum and learning environment

The detailed content of the questionnaire is shown in Table 1.

**Table 1:** Questionary contents to medical students just before graduation.

	Theme	Content
Theme 1	Subjective academic accomplishment	General Education Basic Medicine Social Medicine Clinical Medicine Clinical Clerkship
Theme 2	Length of each curriculum	General Education Basic Medicine Social Medicine Clinical Medicine Clinical Clerkship
Theme 3	Timing to prepare study for summative test	General Education Basic Medicine Social Medicine Clinical Medicine Compute-based Testing (CBT) Pre-CC OSCE Post-CC OSCE National Examination for Doctors
Theme 4	Satisfaction of medical school life and accomplishment	Total Satisfaction of Medical Shool Life Satisfaction of Curriculum Accomplishment Satisfaction of Extracurricular Activities Satisfaction of School Friends

The questionnaire’s content was evaluated by three professionals in medical education. Subsequently, a pilot test involving three medical clerks from our department was conducted.

For evaluating theme 2, 3, a five-point Likert scale (Theme 2: 5 = too long to 1 = too short, Theme 3; 5 = too early to 1 = too late). Medical students rated their confidence using a Visual Analog Scale (VAS), ranging from 0 mm (indicating extreme lack) to 100 mm (indicating an extremely high level) for theme 1 and theme 4. The content of the questionnaire was evaluated by three medical education professionals. A pilot test was then performed by four medical clerks in our center. The online survey was conducted in Japanese using Google Form™ (Google LLC, California, U.S.A.) over a one-week period (from February 20 to February 27, 2024).

### Statistical analysis

Statistical analyses were performed using JMP Pro version 13.2.1 software (SAS Institute Inc., Cary, NC, USA). The results were compared using the Kruskal-Wallis test accompanied by Scheffe’s multiple comparison procedure, chi-square test, and Pearson’s correlation test [17,18]. *P* values <  0.05 were considered statistically significant.

## Results

**Fig 2:**
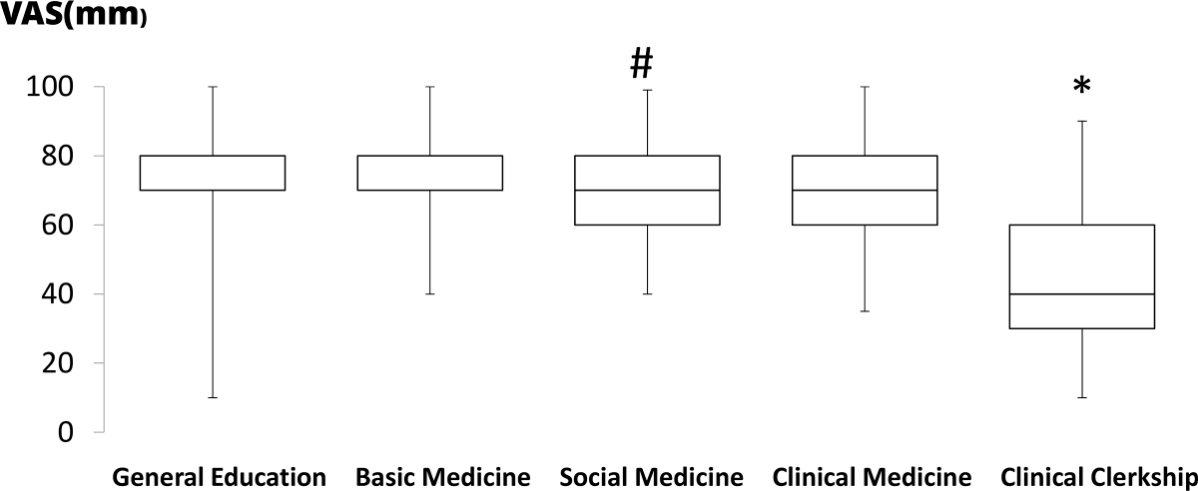
Box-and-whisker plot (median, interquartile range, and range) of confidence toward accomplishment on each medical curricum in students using visual analog scale (VAS), which ranged from 0 mm (extremely unconfident) to 100 mm (extremely confident). * P < 0.05 was considered statistically significant compared to other 4 factors. #P < 0.05 was considered statistically significant compared to basic medicine.

**Fig 3:**
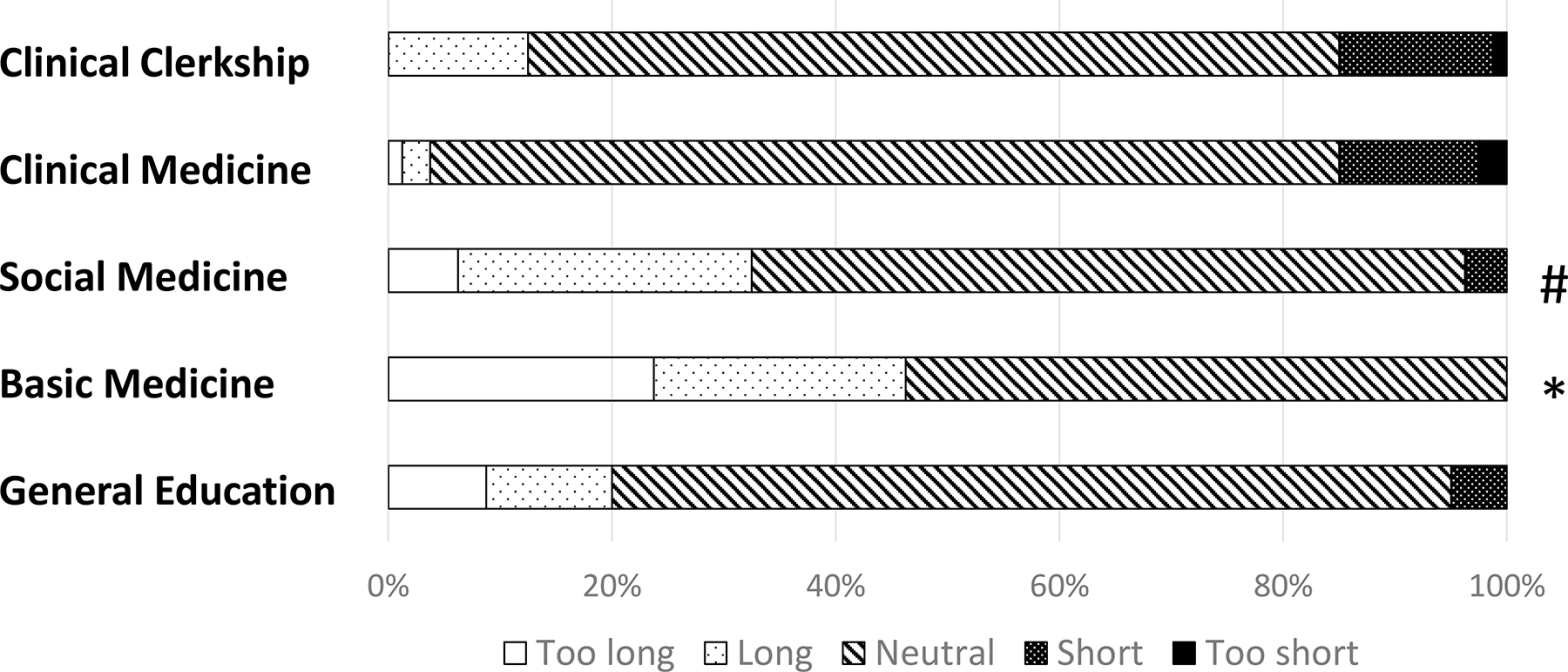
Attitudes toward span of each curriculum (Likert scale: 5 = too long, 4 = long, 3 = neutral, 2 = short, 1 = too short). * P < 0.05 was considered statistically significant. * P < 0.05 was considered statistically significant compared to general education, clinical medicine, and clinical clerkship. #P < 0.05 was considered statistically significant compared to clinical medicine and clinical clerkship.

**Fig 4:**
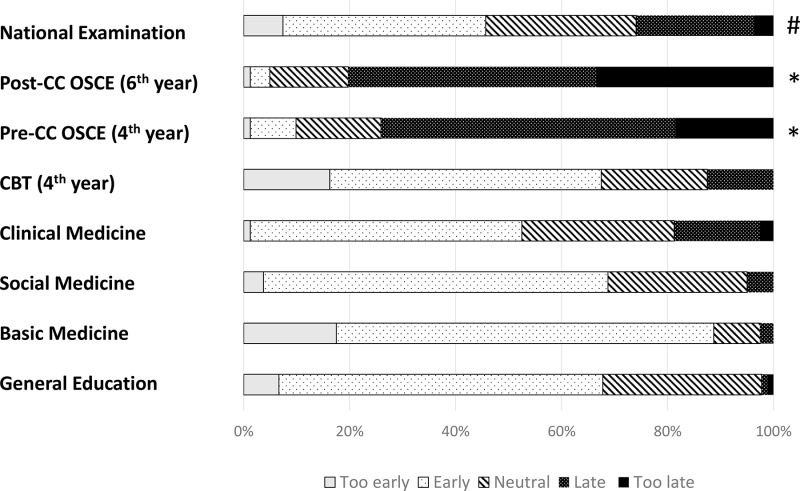
Attitudes toward start time for study toward various examination (Likert scale: 5 = too early, 4 = early, 3 = neutral, 2 = late, 1 = too late). * P < 0.05 was considered statistically significant. * P < 0.05 was considered statistically significant compared to general medicine, basic medicine, clinical medicine, CBT, and clinical clerkship. #P < 0.05 was considered statistically significant compared to general education and basic medicine.

**Fig 5:**
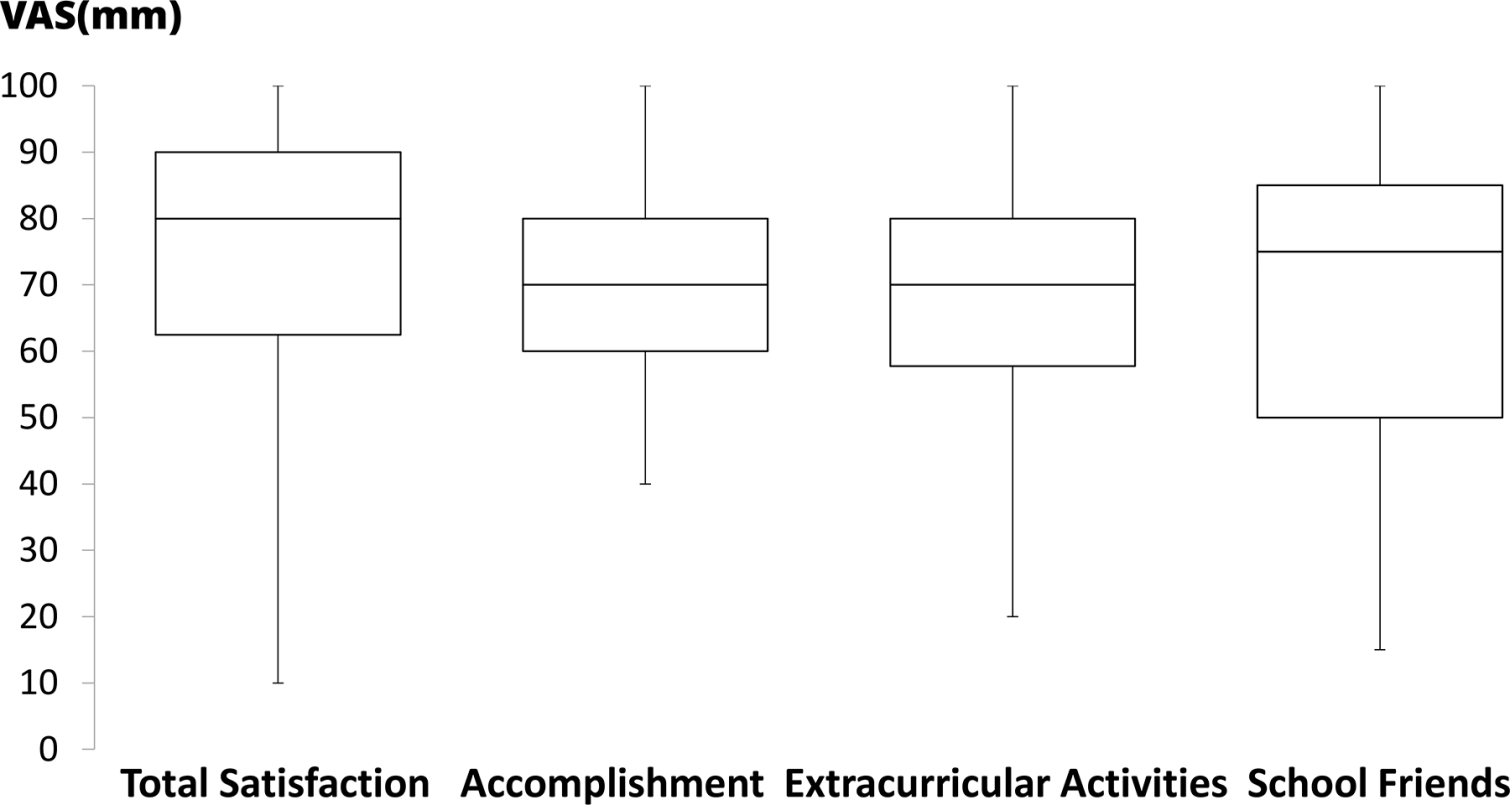
Box-and-whisker plot (median, interquartile range, and range) of satisfaction toward medical school life in students using visual analog scale (VAS), which ranged from 0 mm (extremely unconfident) to 100 mm (extremely confident).

In total, 80 out of 118 6th-year medical students graduating from medical school responded to the survey (response rate: 67.8%). The attitudes toward accomplishment in various curricula are shown in Fig 2. The accomplishment in CC was significantly lower compared to other curricula (P <  0.05, each). Furthermore, the subjective accomplishment in social medicine was significantly lower compared to basic medicine (P =  0.040). Attitudes toward the duration of each curriculum are shown in Fig 3. Graduating students indicated that the subjective duration of basic medicine is significantly longer compared to clinical medicine, CC, and general education (P <  0.05, each). They also indicated that the duration of social medicine was significantly longer than that of clinical medicine and CC (P <  0.05, each). Attitudes toward the timing to start studying for several summative examinations are shown in Fig 4. The subjective timing of preparing for both Pre-CC and Post-CC OSCE was significantly later compared to other summative tests (P <  0.05, each). The subjective timing of the national exam for doctors was significantly later compared to general education and basic medicine (P <  0.05). Attitudes toward summative satisfaction with various aspects of medical school are shown in Fig 5. There was no significant difference among the factors. The correlations among total satisfaction, academic accomplishment, extracurricular activity, and school friends are shown in Table 2. The total satisfaction of medical school life significantly correlated with school friend relationships (P <  0.001). In contrast, there were no significant differences among the other factors.

**Table 2:** Correlation analysis of medical students’ satisfaction in their medical school life. * P < 0.05 was considered significantly different.

	Total Satisfaction	Accomplishment	Extracurricular Activities	School Friends
Total Satisfaction		-0.138 (P = 0.112)	-0.141 (P = 0.106)	0.455 (P < 0.001*)
Accomplishment			-0.105 (P = 0.176)	-0.156 (P = 0.081)
Extracurricular Activities				0.076 (P = 0.251)
School Friends				

## Discussion

In our study, from the viewpoint of curriculum improvement, further skill training is warranted before and during CC, accompanied by clinical knowledge. The most medical students showed they have a weaker sense of accomplishment in CC compared to other curriculum disciplines, while they also feel that the curriculum span of CC is generally appropriate. Notably, medical students consider the time to prepare for the Pre-CC OSCE and Post-CC OSCE to be significantly longer compared to other summative evaluations, including CBT. This suggests that they have more confidence in clinical attitudes or techniques compared to knowledge. This tendency also implies that medical students do not exactly understand how to learn clinical skills. Furthermore, their overall results suggest that graduating medical students feel that they have not studied CC compared to clinical medicine, which is mainly based on clinical knowledge, which implies that there are various improvement points in attitude or skill aspects related to clinical regions. The reason of low subjective accomplishment is associated with educational system during CC. Another possibility is that this may be an issue specific to medical students. Students are often hard on themselves and may feel they haven’t achieved much, even when that’s not the case. During CC, they might feel a sense of powerlessness while working alongside actual healthcare professionals.

Furthermore, the result may highlight the inherent significance of friendships and collaboration within the medical school context. The subjective accomplishment during medical school life showed a significant correlation with school friend relationships, while it did not show a correlation with academic achievements. Also, although we anticipated significant correlation between total satisfaction and extracurricular activity, there was no significant analysis. This is partially contributed to the restriction of extracurricular activity during COVID-19 pandemic. As various reports suggest the limitation of remote learning during COVID-19 pandemic [19,20], this also suggest the significance of on-site learning and mutual interaction between learners in medical school. Learning exclusively from home led to drawbacks, including limited communication and mutual support among students [21,22]. With the resumption of face-to-face classes, issues of insufficient relationship building resulted in student conflicts. The experience highlighted the importance of in-person relationships and mentoring. Post-resumption, prioritizing in-person mutual support, especially through ‘academic support mentoring’ for retention students, became crucial. The widespread adoption of new normal learning, including remote learning, has shifted the significance of face-to-face classes and practical training in universities [23,24]. Despite this, direct interaction and information exchange with classmates in medical education remain crucial for active learning. As new learning environments are explored globally, it is essential to recognize the importance of both horizontal and vertical peer relationships and the significance of extracurricular activities in promoting active learning and mutual support [25,26]. Despite the growing emphasis on interdisciplinary education in medical faculties, the authors argue that ‘collaboration within the same profession or team’ remains a prerequisite for effective interdisciplinary collaboration, highlighting the enduring importance of peer relationships in medical school learning.

This study comes with several noteworthy limitations. Firstly, the data is derived from a single institution, potentially limiting its generalizability to medical schools in other nations [27]. Nevertheless, our findings are likely applicable to medical schools in Japan due to the widespread implementation of the Model Core Curriculum for Medical Education across all institutions. The Model Core Curriculum serves as a systematically organized framework that extracts essential components, which all Japanese universities should address when developing their medical education curricula [28]. Secondly, our analysis included both male and female participants and the question does not include an item that identifies them. An analysis considering gender differences could be an intriguing avenue to explore [29]. Lastly, our quantitative analysis on career design was based on a visual analogue scale assessing awareness or attitude toward career design. Future research should consider employing additional quantitative analyses such as interviews or text-mining analyses on portfolios following career design simulation [30,31].

## Conclusion

We performed web-based survey analyzing the attitude toward medical school whole curriculum and school life assessment in Japanese medical students who are just to graduate. Graduating medical students showed lower subjective accomplishment on CC compared to clinical medicine and other curriculums. Furthermore, total medical school life satisfaction showed significant correlation to medical school friend. Our study on graduating students suggests the need of CC content improvement and inherent role of school friend in medical school learning.

## Supporting information

S1 Checklist PLOS One clinical studies checklist. (DOCX)

S1 File Basic data in this study. (XLSX)

## Abbreviations

CBT: Computer based testing
OSCE: Objective Structured Clinical Education
CC: Clinical Clerkship.
